# Sentinel Surveillance Reveals Emerging Daptomycin-Resistant ST736 *Enterococcus faecium* and Multiple Mechanisms of Linezolid Resistance in Enterococci in the United States

**DOI:** 10.3389/fmicb.2021.807398

**Published:** 2022-02-01

**Authors:** Amy S. Gargis, Lori M. Spicer, Alyssa G. Kent, Wenming Zhu, Davina Campbell, Gillian McAllister, Thomas O. Ewing, Valerie Albrecht, Valerie A. Stevens, Mili Sheth, Jasmine Padilla, Dhwani Batra, J. Kristie Johnson, Alison Laufer Halpin, J. Kamile Rasheed, Christopher A. Elkins, Maria Karlsson, Joseph D. Lutgring

**Affiliations:** ^1^Division of Healthcare Quality Promotion, National Center for Emerging and Zoonotic Infectious Diseases, Centers for Disease Control and Prevention, Atlanta, GA, United States; ^2^Goldbelt C6, LLC, Chesapeake, VA, United States; ^3^Division of Tuberculosis Elimination, National Center for HIV/AIDS, Viral Hepatitis, STD and TB Prevention, Centers for Disease Control and Prevention, Atlanta, GA, United States; ^4^Office of the Director, National Center for Emerging and Zoonotic Infectious Diseases, Centers for Disease Control and Prevention, Atlanta, GA, United States; ^5^Biotechnology Core Facility Branch, Division of Scientific Resources, Centers for Disease Control and Prevention, Atlanta, GA, United States; ^6^ASRT Incorporated, Atlanta, GA, United States; ^7^Department of Pathology, University of Maryland School of Medicine, Baltimore, MD, United States

**Keywords:** *Enterococcus faecalis*, *Enterococcus faecium*, daptomycin, linezolid, conjugation, *optrA*, transmission, pheromone responsive plasmid

## Abstract

*Enterococcus faecalis* and *faecium* with resistance to daptomycin and/or linezolid are emerging globally. We present the genomic characterization of daptomycin- and linezolid-resistant *E. faecalis* and *E. faecium* surveillance isolates from the United States, 2013–2016. Daptomycin resistance was low among *E. faecalis* (2/364, 0.5%) and *E. faecium* (17/344, 5%). The majority (71%, 12/17) of daptomycin-resistant *E. faecium* isolates belonged to the emerging ST736 clone and contained mutations in *liaFSR* and *cls* previously associated with resistance. However, 1/2 *E. faecalis* and 3/17 *E. faecium* did not contain these mutations previously associated with daptomycin resistance. Linezolid resistance was rare among *E. faecalis* (1/364, 0.3%) and *E. faecium* (2/344, 0.6%). These two *E. faecium* isolates, one of which was also resistant to daptomycin and vancomycin, contained the 23S rRNA nucleotide mutation (G2576T) associated with linezolid resistance. Long-read sequencing revealed the linezolid-resistant *E. faecalis* isolate contained chromosomal- and plasmid-encoded copies of *optrA*. The chromosomal *optrA* was located on the recently described Tn*6674* multiresistance transposon. The second copy of *optrA* was encoded on an ∼65 kb mosaic plasmid, with component regions sharing high sequence identity to *optrA*-encoding multiresistance plasmids of animal origin. The *optrA*-encoding plasmid contained open reading frames predicted to encode proteins associated with a pheromone-responsive plasmid transfer system, and filter mating experiments confirmed the plasmid was conjugative. Continued surveillance of enterococci is necessary to assess the prevalence and trends of daptomycin and linezolid resistance in the United States, characterize resistance mechanisms and how they transfer, and monitor for emerging sequence types associated with resistance.

## Introduction

Enterococci are an important cause of healthcare-associated infections (HAIs) in the United States, including bloodstream, surgical site, and urinary tract infections. Approximately 30% of enterococcal HAIs are caused by vancomycin-resistant enterococci (VRE), which are categorized as a serious public health threat, requiring prompt and sustained action (CDC, 2019). Enterococci resistant to penicillin, ampicillin, and vancomycin (VAN) require other treatments such as daptomycin (DAP) and linezolid (LZD) (Garcia-Solache and Rice, 2019). However, resistance to these other agents is also being reported (Arias et al., 2011; Pfaller et al., 2017).

Daptomycin is a lipopeptide antibiotic that binds to the bacterial cell membrane (CM), disrupting essential envelope functions and resulting in cell death (Prater et al., 2019). In *Enterococcus faecalis*, daptomycin resistance (DAP-R) is a result of CM changes that redistribute phospholipids away from the septum, diverting DAP from the cell (Khan et al., 2019). In *Enterococcus faecium*, the environment can influence how DAP-R evolves, and both repulsion and DAP diversion mechanisms can occur (Prater et al., 2019). In both *E. faecalis* and *E. faecium*, these CM responses are most commonly associated with initial mutations in genes encoding the 3-component LiaFSR (lipid-II–interacting antibiotics) stress response system that regulate cell envelope integrity, followed by subsequent mutations in genes involved in phospholipid metabolism (Khan et al., 2019), including *cls*, encoding a cardiolipin (CL) synthase; and *gdpD*, encoding a putative glycerophosphodiesterase (Prater et al., 2019). The primary modulator of the LiaFSR system in *E. faecalis*, LiaX, was recently characterized and found to sense DAP and trigger protective cell membrane remodeling (Khan et al., 2019). A recent report identified the first clinical case of an *E. faecalis* isolate with a LiaX loss-of-function mutation resulting in DAP-R (Ota et al., 2021). While the mutations in the LiaFSR system are the most common mechanism associated with DAP-R, resistance is driven by complex changes in the membrane stress response network and additional pathways exist. For example, the YycFG and YxdJK 2-component systems that regulate cell wall homeostasis have also been implicated in enterococcal DAP-R (Miller et al., 2019, 2020). Limited data are available on the clonal distribution of DAP-R enterococcal clinical isolates in the United States, but a clone, ST736, associated with DAP-R has recently been reported in New York City (Wang et al., 2014, 2018).

Linezolid is an oxazolidinone antibiotic effective against Gram-positive bacteria, including enterococci and *Staphylococcus aureus*. In enterococci, resistance to LZD is often caused by mutations in the V domain of the 23S rRNA gene (Hasman et al., 2019), with the G2576T nucleotide mutation (Beukers et al., 2018) being the most common (Bi et al., 2018). Recently, the resistance genes *cfr*, *cfr*(B), *cfr*(D), *optrA*, and *poxtA*, encoding transferable resistance to oxazolidinones, have been described in enterococci (Liu et al., 2012, 2014; Deshpande et al., 2015; Wang et al., 2015; Bender et al., 2016; Antonelli et al., 2018; Guerin et al., 2020). The *optrA* gene encodes an ATP-binding cassette (ABC)-F protein that mediates resistance through the ribosomal protection mechanism (Sharkey et al., 2016). First characterized in *E. faecalis* and *E. faecium* isolated in China from humans and food-producing animals in 2009 (Wang et al., 2015), *optrA* has since been identified on the chromosome or on various-sized plasmids, sometimes in combination with *cfr*, *cfr*(B), or *poxtA* within the same strain or plasmid (Li et al., 2019). When encoded on the chromosome, *optrA* is typically located on a recently characterized Tn*554*-family transposon, Tn*6674* (Li et al., 2019), adjacent to the resistance gene *fexA*, which confers resistance to phenicols (Freitas et al., 2020). Tn*6674* insertion occurs at a conserved location in the *radC* gene, which encodes a putative DNA repair protein, and this Δ*radC* integration site is considered a hotspot for chromosomal *optrA* integration (Freitas et al., 2020). When encoded on a plasmid, the *optrA* gene is typically flanked (upstream and/or downstream) by insertion sequence elements of the IS*1216* (IS*6* family) or ISL*3* family type (He et al., 2016; Freitas et al., 2020). The *optrA* gene has rapidly emerged as a major contributor to the spread of LZD resistance worldwide (Deshpande et al., 2018), but has been only recently detected in human clinical (Pfaller et al., 2017; Deshpande et al., 2018) and animal isolates (Tyson et al., 2018) in the United States. Limited data are available regarding prevalence of *optrA* among clinical or animal linezolid- resistant (LZD-R) enterococcal isolates in the United States; however, the available studies indicate enterococci harboring *optrA* remain low (Pfaller et al., 2017; Deshpande et al., 2018; Tyson et al., 2018; Wardenburg et al., 2019). For example, a recent study of United States (U.S.) clinical *E. faecium* LZD-R isolates revealed they were more likely to harbor mutations in the V domain of the 23S rRNA gene than to carry *optrA* (Wardenburg et al., 2019).

The U.S. Centers for Disease Control and Prevention’s (CDC), Division of Healthcare Quality Promotion (DHQP) Sentinel Surveillance system performs surveillance of isolates collected from geographically diverse regions of the United States to study their antimicrobial resistance profiles and perform phenotype-genotype correlation studies. The goal of this surveillance is to improve the knowledge of susceptibility patterns and mechanisms of resistance among bacteria causing HAIs. Isolates are collected from patients hospitalized in acute care or long-term care facilities. Here, we present the characterization of *E. faecalis* and *E. faecium* isolates collected during the DHQP Sentinel Surveillance system’s 2013–2016 collection period determined to be DAP-R or LZD-R by reference antimicrobial susceptibility testing (AST). Whole genome sequencing (WGS) was used to identify if there were predominant multi-locus sequence typing (MLST) clones associated with DAP-R or LZD-R, and if mechanisms or mutations previously associated with DAP-R or LZD-R could be identified.

## Materials and Methods

### Bacterial Isolates, Growth Conditions, and Identification

All isolates included in this study were selected from a Sentinel Surveillance collection of 708 enterococci collected during 2013–2016 from eight states (CA, IA, MD, NC, NM, NY, PA, and WA) and resistance to DAP and/or LZD was determined by antimicrobial susceptibility testing (AST) (described below). Isolates were cultured on BD BBL Trypticase soy agar II with 5% sheep blood (SBA) (Thermo Fisher Scientific, Waltham, MA, United States) at 37°C in ambient air from glycerol stocks stored at −70°C. Identification was confirmed with matrix-assisted laser desorption/ionization-time of flight (MALDI-TOF) mass spectrometry using a MALDI Biotyper, version 3.1 (Bruker Daltronics, Bremen, Germany).

### Antimicrobial Susceptibility Testing

Broth microdilution (BMD) AST was performed on all isolates following the Clinical and Laboratory Standards Institute (CLSI) guidelines (CLSI, 2018, 2021). BMD panels were prepared in-house, consisting of 14 antibiotics at varying twofold serial dilutions in sterile 96-well microtiter plates (Caplugs, Rancho Dominguez, CA, United States) and stored at −70°C until use. Each well contained 100 μl Cation-adjusted Mueller-Hinton Broth (CAMHB, BD Difco, Sparks, MD, United States) and for DAP there was a supplement of 50 μg/ml calcium chloride. The following antimicrobial concentrations were included in the BMD panels: DAP (0.25–16 μg/ml), LZD (0.5–16 μg/ml), and VAN (0.25–64 μg/ml), see Supplementary Table 1 for the full list of antibiotics and concentrations tested in the BMD panel. *Enterococcus faecalis* ATCC 29212 and *Staphylococcus aureus* ATCC 29213 were used as quality control strains. Samples and controls were streaked to purity on SBA and colonies were suspended in 5 ml REMEL 0.85% sterile saline (Thermo Fisher Scientific, Waltham, MA, United States) to a turbidity equal to a 0.5 McFarland standard. Panels were inoculated with a 95-pin sterile inoculator (10 μl pickup; Caplugs, Buffalo, NY, United States), incubated at 35°C and read and interpreted according to CLSI guidelines (CLSI, 2021).

### Whole Genome Sequencing

All DAP-R or LZD-R isolates underwent WGS. Genomic DNA was extracted from colonies cultured overnight on SBA using the Promega Maxwell^®^ 16 Cell Low Elution Volume DNA Purification Kit and Maxwell^®^ 16 MDx Instrument (Madison, WI, United States) and sheared using the Covaris ME220 Focused-ultrasonicator*™* (Woburn, MA, United States). Indexed libraries were prepared using the Tecan Ovation^®^ Ultralow System V2 Assay Kit (San Carlos, CA, United States) and the PerkinElmer Zephyr^®^ G3 NGS Workstation (Waltham, MA, United States). Libraries were analyzed using the Standard Sensitivity NGS Fragment Analysis Kit and Advanced Analytical Fragment Analyzer System (Ankeny, IA, United States). Sequencing was performed using the MiSeq^®^ Reagent Kit v2 (500 cycle) and Illumina MiSeq^®^ System (San Diego, CA, United States) generating 2 × 250 paired-end reads. The Wizard^®^ Genomic DNA Purification Kit (Promega, Madison, WI, United States) was used to extract high molecular weight DNA from the *optrA*-positive *E. faecalis* AR-0780 following manufacturer’s instructions. DNA sheared to 20 kb utilizing needle shearing was used to generate large SMRTbell libraries using the standard 20-kb library protocols of the Pacific Biosciences SMRTbell template prep kit 1.0 (PacBio, Menlo Park, CA, United States). The libraries were further size selected utilizing BluePippin (Sage Scientific, Beverly, MA, United States) with a cutoff size of 10 kb. The finished library was bound to proprietary P6v2 polymerase and sequenced on a PacBio RS II sequencer using C4v2 chemistry for 360-min movies. PacBio reads were assembled *de novo* with HGAP (v3). Assemblies were polished with PacBio reads using Quiver, then polished with Illumina reads using Pilon (v 1.22) (Chin et al., 2013; Walker et al., 2014; Hunt et al., 2015). Sequence data were analyzed using the QuAISAR-H pipeline; a description of custom scripts and publicly available tools and versions utilized by QuAISAR-H is available at https://github.com/DHQP/QuAISAR_singularity. Sequence types (STs) were assigned using the pubMLST *E. faecalis* or *E. faecium* multi-locus sequence typing schemes (Homan et al., 2002; Ruiz-Garbajosa et al., 2006; Jolley et al., 2018) and clonal complexes (CCs) were assigned using the pubMLST database or as previously described in the literature, see below. Antimicrobial resistance genes were identified using SRST2 (v0.2.0) (Inouye et al., 2014) and GAMMA (Stanton et al., 2021) against a non-redundant combined database of the AR databases ResFinder (last updated 10/1/2019) (Zankari et al., 2012), ARG-ANNOT (v6, July 2019) (Gupta et al., 2014), and NCBI’s AMRFinder (last updated 11/5/2019) (Feldgarden et al., 2019) (all accessed 12/27/2019); minimum of 98% sequence identity and 90% sequence coverage was used. Assembled genomes were submitted to the NCBI Prokaryotic Genome Annotation Pipeline for annotation. An *E. faecium* whole genome multi-locus sequence typing (wgMLST) schema consisting of 5,489 accessory loci was used to analyze the ST739 isolates (BioNumerics, v7.6).^1^ The wgMLST phylogeny was constructed using Interactive Tree Of Life (iTOL, v5) (Letunic and Bork, 2021). To compare plasmids, assemblies were annotated with Prokka (v1.12) (Seemann, 2014) for consistency, aligned with NCBI Blast (v2.2.22) (Camacho et al., 2009), and visualized with EasyFig (v2.2.2) (Sullivan et al., 2011). CGE PlasmidFinder 2.0 was used to type pAR-0780 (Carattoli et al., 2014).

### Analysis of Whole Genome Sequencing Data

Analysis of nucleotide mutations associated with LZD resistance within the 23S rRNA gene were performed using the recommendations provided by Beukers et al. (2018). Briefly, the 23S rRNA gene from *E. faecium* strain Aus0004 (GenBank accession number: NR_103056) was used as the reference sequence and each sample’s reads were mapped to the reference gene using Bowtie 2 (Langmead and Salzberg, 2012), then processed using SAMTools (Li et al., 2009) and then SNP-called using VarScan (v2.4.1) (Koboldt et al., 2012) using mpileup2cns with the following settings: –min-coverage 8 –min-reads2 2 –min-var-freq 0.01. WGS data from the LZD-R isolates were also analyzed for the detection of 23S rRNA mutations, and the *optrA*, *cfr*, *cfr*(B) and *poxtA* genes using LRE-finder 1.0^2^ (Hasman et al., 2019). Analysis of assemblies for the presence of mutations associated with DAP resistance and analysis of the *optrA* regions on AR-0780’s chromosome and plasmid were performed using CLC Genomics Workbench 11.^3^ The *liaF*, *liaS, liaR, gdpD*, and *yycG* gene sequences from the *E. faecium* strain DO (accession CP003583), and the *cls* gene sequence from the *E. faecium* strain UW7606 × 64/3 TC1 (accession CP013009) were used to identify mutations in study strains as described by Wang et al. (2018). *E. faecalis*, strain S316 (accession ADDP01000082) was used to identify mutations in *liaF*, *liaS, liaR, cls*, and *gdpD* coding regions. The *liaX* coding region from *E. faecium* strain R494 (accession JH807905) and *E. faecalis* strain V583 (accession AE016830) were used to identify *liaX* mutations. The *E. faecium* HOU503 (accession CP040706) strain’s *pgsA* coding region was used as a reference to identify mutations in *E. faecium* study strains.

### Conjugation Experiments

Filter mating experiments were performed to assess the transferability of the *E. faecalis* AR-0780 plasmid, pAR-0780, that encodes *optrA*. Isolated colonies of the donor, *E. faecalis* AR-0780 (*optrA* +, LZD-resistant, fusidic acid-susceptible) and the recipient *E. faecalis* JH2-2 (fusidic acid-resistant, LZD-susceptible), were inoculated into 5 ml of BD BBL Myoflask Trypticase Soy Broth (TSB, BD, Franklin Lakes, NJ, United States) containing 5 μl LZD (25 μg/ml) or 5 μl fusidic acid (3 μg/ml), respectively, and incubated overnight at 37°C. One hundred microliters of each overnight culture were transferred to 5 ml TSB, vortexed briefly, and incubated at 37°C for ∼5 h to mid-log phase. The turbidity of each culture was adjusted to the same optical density in sterile 0.85% saline (REMEL, Thermo Fisher Scientific, Waltham, MA, United States) using a Dade Behring Microscan turbidity meter (Siemens, Munich, Germany). Twenty microliters of the AR-0780 suspension was mixed with 180 μl of JH2-2 for a 1:10 ratio of donor to recipient. The conjugation mixture was applied to the center of a Corning 0.22-μm pore bottle top filter (Thermo Fisher Scientific, Waltham, MA, United States) and collected by vacuum filtration. The filter was removed from the filter unit, placed in the middle of a SBA plate, without antimicrobials, and incubated at 37°C for 24 h. The filter was then transferred to a sterile 50 ml conical tube containing 5 ml 0.85% saline and vortexed vigorously to remove growth. Serial dilutions of the conjugation mixture were prepared in sterile 0.85% saline and 100 μl of each dilution were plated in triplicate onto trypticase soy agar (TSA, BD) plates containing the following antimicrobial agents for selection: TSA with 3 μg/ml LZD (donor selection); TSA with 25 μg/ml fusidic acid (recipient selection); and TSA with 3 μg/ml LZD and 25 μg/ml fusidic acid (transconjugant selection). Following incubation at 37°C for 36–48 h, donor, recipient, and transconjugant colonies were counted to calculate the conjugation frequency (transconjugants per donor). Ten transconjugant colonies were randomly selected and propagated on TSA plates containing 3 μg/ml LZD and 25 μg/ml fusidic acid. PCR was performed to verify the presence of *optrA* in the transconjugants as described above.

### *optrA* PCR

A cell lysate was prepared (Conrad et al., 1996) and used for PCR to verify the presence of *optrA* as previously described (Wang et al., 2015), using primers A-F (5′-AGGTGGTCAGCGAACTAA-3′) and A-R (5′-ATCAACTGTTCCCATTCA-3′) that amplify a 1,395 bp internal segment of *optrA*. PCR reaction mixtures consisted of 25 μl HotStarTaq Master Mix (QIAGEN, Germantown, MD), 1 μl of each primer (20 μM), 18 μl RNAse-free water (Promega, Madison, WI, United States), and 5 μl of each cell lysate. PCR reaction conditions were 95°C 15 min; 35 cycles of 94°C 1 min, 55°C 1 min, 72°C 1 min; and a final extension of 72°C for 7 min.

### Confirmation of Plasmid Transfer by S1 Nuclease-Pulsed-Field Gel Electrophoresis

Two transconjugants that were PCR-positive for *optrA* and donor and recipient controls were analyzed by S1 nuclease-pulsed-field gel electrophoresis (PFGE) as previously described,^4^ with the following modifications for S1 nuclease digestion. Agarose plug slices were cut approximately 1.5 mm wide and digested in a reaction mix consisting of 134 μl molecular grade water, 15 μl l0X restriction buffer (Takara Bio, Mountain View, CA, United States), and 20U S1 nuclease (Takara Bio, Mountain View, CA, United States). Samples were incubated for 30 min at 37°C, then subjected to contour-clamped homogenous electric field (CHEF) PFGE using the CHEF XA system (Bio-Rad Laboratories, Hercules, CA). The MidRange PFG Marker (New England Biolabs, Inc., Ipswich, MA, United States) was included as a size marker. Electrophoresis was carried out at 6 V/cm, 14°C for 20 h, 120° included angle, with switch times of 1 and 25 s. The gel was stained in reverse osmosis water containing GelRed^®^ (Biotium, Fremont, CA, United States) at 1:10,000 and photographed using a UV photoillumination system (Analytik Jena, Jena, Germany).

## Results

### Antimicrobial Susceptibility of Daptomycin- or Linezolid-Resistant Isolates

During the 2013–2016 isolate collection period, 708 enterococci, identified as *E. faecalis* (*n* = 364) or *E. faecium* (*n* = 344) by MALDI-TOF, were tested using BMD AST. Nineteen isolates, (2/364, 0.5% *E. faecalis* and 17/344, 4.9%, *E. faecium*) were DAP-R, with MICs ranging from 8 to > 16 μg/ml (Table 1). Three isolates, 1/364 (0.3%) *E. faecalis* and 2/344 (0.6%) *E. faecium*, were LZD-R, with MICs ranging from 8 to 16 μg/ml, including one *E. faecium* isolate also resistant to DAP and VAN (Table 2). All three *E. faecalis* isolates included in this study were VAN-S, while 10/18 *E. faecium* isolates were VAN-R and eight were VAN-S (Tables 1, 2 and Supplementary Table 1). The full antimicrobial susceptibility profiles for all isolates and antimicrobials tested are summarized in Supplementary Table 1.

**TABLE 1:** Characteristics of *E. faecalis* and *E. faecium* CDC Sentinel Surveillance isolates with daptomycin resistance.

Isolate ID	BioSample	Organism ID	MLST^a^	MLST CC/Clade	VAN MIC μg/ml	VAN Int^b^	DAP MIC μg/ml	DAP Int^b^	DAP-R mutations*^c^*
1603244	SAMN16584113	*E. faecalis*	ST1052^a^	N/A	1	S	16	R	LiaF^Ins I177^
1603442	SAMN16584119	*E. faecalis*	ST40	CC40	2	S	8	R	N/A
1603019	SAMN16584109	*E. faecium*	ST736	CC17/A1	>64	R	16	R	LiaR^W73C^, LiaS^T120A,^ Cls^N13S^
1603296	SAMN16584116	*E. faecium*	ST736	CC17/A1	>64	R	16	R	LiaR^W73C^, LiaS^T120A,^ Cls^H215R^
1603630	SAMN16584123	*E. faecium*	ST736	CC17/A1	1	S	8	R	LiaR^W73C^, LiaS^T120A,^ Cls^H215R^
1704051	SAMN16584126	*E. faecium*	ST736	CC17/A1	>64	R	16	R	LiaR^W73C^, LiaS^T120A^, Cls^R211Q^
1603515	SAMN16584122	*E. faecium*	ST736	CC17/A1	>64	R	16	R	LiaR^W73C^, LiaS^T120A^, Cls^A20P^
1603084	SAMN16584110	*E. faecium*	ST736	CC17/A1	>64	R	8	R	LiaR^W73C^, LiaS^T120A^
1603243	SAMN16584112	*E. faecium*	ST736	CC17/A1	>64	R	8	R	LiaR^W73C^, LiaS^T120A^
1603275	SAMN16584115	*E. faecium*	ST736	CC17/A1	1	S	8	R	LiaR^W73C^, LiaS^T120A^
1603468	SAMN16584120	*E. faecium*	ST736	CC17/A1	>64	R	8	R	LiaR^W73C^, LiaS^T120A^
1603514	SAMN16584121	*E. faecium*	ST736	CC17/A1	1	S	8	R	LiaR^W73C^, LiaS^T120A^
1603688	SAMN16584125	*E. faecium*	ST736	CC17/A1	1	S	8	R	LiaR^W73C^, LiaS^T120A^
1704054	SAMN16584127	*E. faecium*	ST736	CC17/A1	1	S	8	R	LiaR^W73C^, LiaS^T120A^
1603162	SAMN16584111	*E. faecium*	ST17	CC17/A1	>64	R	>16	R	Cls^V37A, R267S^
1603274	SAMN16584114	*E. faecium*	ST18	CC17/A1	>64	R	8	R	Cls^R211Q^
1704095	SAMN16584128	*E. faecium*	ST78	CC17/A1	>64	R	8	R	N/A
1603637	SAMN16584124	*E. faecium*	ST1840^a^	CC94	1	S	8	R	N/A
1603389	SAMN16584118	*E. faecium*	ST1841^a^	N/A	1	S	8	R	N/A

*^a^Sequence types that were previously unknown and submitted to the pubMLST database as part of this work.*

*^b^Interpretive criteria were applied according to CLSI performance standards for antimicrobial susceptibility testing M100 (CLSI, 2021).*

*^c^Amino acid mutations in LiaR, LiaS, and Cls are designated. The Cls mutation not previously reported as associated with DAP resistance is underlined. MLST, multi-locus sequence type (ST); CC, clonal complex; VAN, vancomycin; DAP, daptomycin; MIC, minimal inhibitory concentration; N/A, no mutations present.*

**TABLE 2:** Characteristics of *E. faecalis* and *E. faecium* CDC Sentinel Surveillance isolates with linezolid resistance.

Isolate ID	BioSample	Organism ID	MLST	MLST CC	VAN MIC μg/ml	VAN Int^a^	DAP MIC μg/ml	DAP Int^a^	LZD MIC μg/ml	LZD Int^a^	LZD-R mutation/gene identified^b^
AR-0780	SAMN11953790	*E. faecalis*	ST256	CC256	1	S	1	S	8	R	*optrA*
1603243	SAMN16584112	*E. faecium*	ST736	CC17	>64	R	8	R	16	R	23S rRNA, G2576T
1603370	SAMN16584117	*E. faecium*	ST736	CC17	0.5	S	4	SDD	8	R	23S rRNA, G2576T

*^a^Interpretive criteria were applied according to CLSI performance standards for antimicrobial susceptibility testing M100 (CLSI, 2021).*

*^b^The gene or nucleotide mutation associated with linezolid resistance is designated. MLST, multi-locus sequence type (ST); CC, clonal complex; VAN, vancomycin; DAP, daptomycin; LZD, linezolid; MIC, minimal inhibitory concentration; SDD, susceptible dose dependent.*

### Whole Genome Sequence Analysis of Daptomycin-Resistant Enterococci

Whole genome sequencing was performed on all isolates and the resulting assemblies were used to determine MLSTs. Of the two DAP-R *E. faecalis* isolates, one had an unknown ST. The novel profile was submitted to the pubMLST database^5^ and assigned ST1052 but did not have an associated CC (Table 1). This isolate contained an amino acid insertion, LiaF^Ins I177^, previously associated with DAP-R (Miller et al., 2013). The other DAP-R *E. faecalis* isolate, 1603442, was ST40, CC40 (Mikalsen et al., 2015), and did not contain mutations previously associated with DAP-R in *liaF*, *liaS liaR, liaX, cls*, or *gdpD*.

Of the 17 DAP-R *E. faecium* isolates, two had unknown STs and the novel profiles were submitted to the pubMLST database^6^ and assigned ST1840, which belonged to CC94, and ST1841, which did not have an associated CC (Table 1). Twelve of 17 (71%) *E. faecium* isolates were ST736 (CC17/Clade A1) (Wang et al., 2018; Top et al., 2020). The remaining three *E. faecium* isolates were ST17, ST18, and ST78, which also belong to CC17/Clade A1.

**FIGURE 1:**
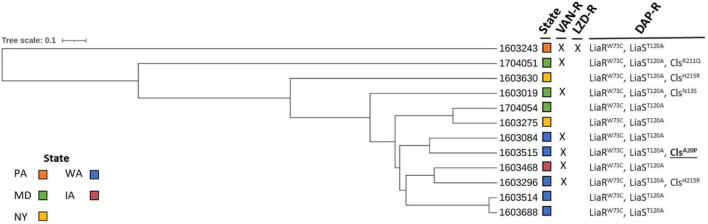
Whole genome multi-locus sequence typing of daptomycin-resistant ST736 *E. faecium* isolates. Vancomycin-resistant (VAN-R), linezolid-resistant (LZD-R), and daptomycin-resistant (DAP-R) profiles are indicated (X: resistant). Amino acid mutations in LiaR, LiaS, and Cls are designated (Cls mutation not previously reported as associated with DAP resistance is underlined and in bold). States from which the isolates were collected are indicated (colored boxes).

All 12 ST736 *E. faecium* isolates contained the LiaR^W73C^ and LiaS^T120A^ amino acid mutations (Table 1) previously associated with DAP-R ST736 isolates (Wang et al., 2018). Five ST736 isolates also contained mutations in Cls, including those previously associated with DAP-R, Cls^N13S^ (1603019), Cls^H215R^ (1603296 and 1603630), and Cls^R211Q^ (1704051) (Wang et al., 2018; Prater et al., 2021). ST736 isolate 1603515 contained an alanine to proline substitution in Cls, Cls^A20P^, not previously associated with DAP-R. Seven of the 12 (58%) DAP-R ST736 isolates were also VAN-resistant, and all contained *vanA*. Whole genome MLST was performed to assess the diversity of ST736 *E. faecium* isolates with respect to state collected, AST and DAP-R mutation profile, and the resulting phylogeny is provided in Figure 1. DAP-R ST736 isolates were collected from WA (*n* = 5), MD (*n* = 3), NY (*n* = 2), IA (*n* = 1), and PA (*n* = 1).

The five non-ST736 *E. faecium* DAP-R isolates did not contain the LiaR^W73C^ and LiaS^T120A^ mutations seen in all DAP-R ST736 isolates. Isolate 1603274 (ST18, CC17), contained the Cls^R211Q^ seen in 1704051 (ST736) that was previously associated with DAP-R (Prater et al., 2021). Isolate 1603162 (ST17) contained two mutations in Cls (Cls^V37A, R267S^) previously associated with DAP-R (Palmer et al., 2011; Prater et al., 2021), but did not contain mutations in LiaF, LiaS, or LiaR. The remaining three *E. faecium* DAP-R isolates (1603637/ST1840, 1603389/ST1841, and 1704095/ST78) did not contain mutations previously associated with DAP-R in the genes analyzed.

### Whole Genome Sequence Analysis of Linezolid-Resistant Enterococci

**FIGURE 2:**
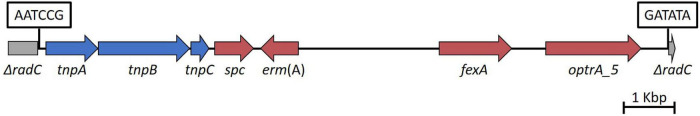
Diagram of AR-0780’s chromosomal transposon, Tn*6674* (12,933 bp). *optrA* along with the resistance genes *spc*, *erm*(A), and *fexA*, are shown in red. Transposase genes, *tnpA, tnpB, and tnpC*, are shown in blue. The left and right hexanucleotide sequences marking the left and right junction of the Tn*6674 radC* integration site are shown in boxes. The disrupted *radC* (Δ*radC*) is shown in gray.

Whole genome sequencing data were assessed to identify the presence of genes and mutations known to be associated with LZD resistance, including screening for the presence of *cfr* and *cfr* variants, *optrA*, *poxtA*, and mutations in the 23S rRNA gene. The LZD-R ST736 *E. faecium* isolates, 1603243 (DAP-R and VAN-R) and 1603370 (DAP-susceptible dose dependent, and VAN-S) contained the 23S rRNA G2576T nucleotide mutation previously associated with resistance (Table 2; Prystowsky et al., 2001; Beukers et al., 2018). The only LZD-R *E. faecalis* isolate identified, AR-0780, was ST256 (CC256) (Wang et al., 2018) and contained *optrA* (Table 2). To analyze the genetic environment of *optrA*, AR-0780 was sequenced using both short-read (MiSeq) and long-read (PacBio) technologies to create an annotated hybrid assembly. The complete genome consisted of a 2.7 Mb circular chromosome and a single 65,096 bp plasmid, designated pAR-0780. There were two distinct copies of *optrA* identified, one on the chromosome and one on the plasmid. The *optrA* encoded on the chromosome contained two nucleotide variants, G1779A (a synonymous mutation at position K595K) and C1833T (a synonymous mutation at position I611I), that correspond to the *optrA* variant designated as *optrA_5* in the Center for Genomic Epidemiology’s database (see text footnote 2, accessed January 2021) (Hasman et al., 2019). An NCBI BLASTN search using the chromosomal *optrA* region of isolate AR-0780 as a query sequence identified a 12,933 bp region with 100% query coverage and ≥ 99.8% nucleotide sequence identity with other chromosomal *optrA* gene clusters in GenBank, including *E. faecalis* strains A101 (MH018572.1), TZ2 (MH225421.1), C25 (MK251150.1), and E1731 (MK737778), which were recently characterized and designated Tn*6674*, a novel *optrA*-carrying multiresistance transposon of the Tn*554* family/Group I (Li et al., 2019; Freitas et al., 2020). This transposon carries the *optrA* gene, as well as the spectinomycin resistance gene *spc*, the chloramphenicol and florfenicol resistance gene *fexA*, and the macrolide-lincosamide-streptogramin B resistance gene *erm*(A) (Figure 2). The Tn*6674* transposon was inserted into the *radC* gene, which is a common integration site for Tn*554*-family transposons (Li et al., 2019; Freitas et al., 2020). The boundaries of AR-0780’s Tn*6674* were defined by hexanucleotide sequences at the left (5′-AATCCG-3′) and right (5′-GATATA-3′) junction, which were similar to junction sequences of other Tn*554*-family transposons (Li et al., 2019).

**FIGURE 3:**
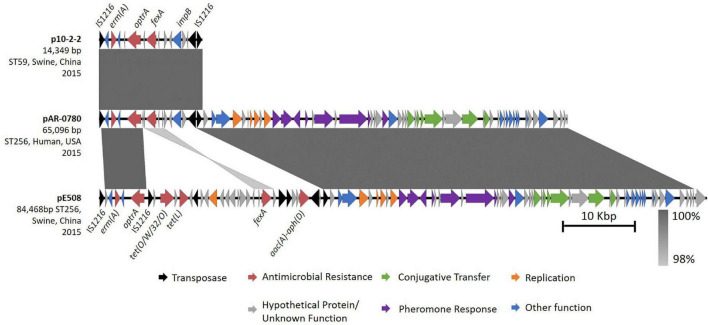
Comparative analysis of pAR-0780 with similar *optrA*-encoding plasmids. pAR-0780 contained a near identical copy (100% query coverage, 99.95% identity) of a 14,349 bp *optrA* gene cluster flanked on the left- and right-hand sides by IS*1216*R elements (black) previously described on plasmid p10-2-2 from an *E. faecalis* isolate (ST59) recovered from a pig in China in 2015. The remaining 51,543 bp of pAR-0780 was nearly identical (100% coverage, 99.88% identity) to a 51,543 region of the 84,468 bp plasmid, pE508, a pheromone-responsive conjugative multiresistance plasmid from *E. faecalis* strain E508 (ST256), isolated from a swine fecal sample in 2015.

The second copy of *optrA*, encoded on pAR-0780 was unique from the chromosomally encoded *optrA_5*, and contained one mutation at nucleotide position A1441C (non-synonymous mutation, T481P). The position of this mutation has been described before (Freitas et al., 2020) in *optrA* alleles, but only in isolates containing additional *optrA* mutations; therefore, no variant designation was available in the Center for Genomic Epidemiology’s database (Hasman et al., 2019). BLASTN analysis of the regions flanking the plasmid-encoded *optrA* revealed pAR-0780 contained a near identical (100% query coverage, 99.95% nucleotide sequence identity) 14,349 bp *optrA* gene cluster flanked on the left- and right-hand sides by IS*1216* elements in the same orientation previously described on a fragment of an *optrA*-encoding plasmid p10-2-2 (KT862775.1) from an *E. faecalis* isolate (ST59) recovered from a pig in China (He et al., 2016; Figure 3). The *optrA* gene encoded on p10-2-2 contains two nucleotide variants: the A1441C (non-synonymous mutation, T481P) variant seen in pAR-0780 and the T526G (non-synonymous mutation, D176Y), which correspond to *optrA_7* in the Center for Genomic Epidemiology’s database. The pAR-0780 plasmid contained the *impB-fexA-optrA* backbone that is common in Group II *optrA* positive isolates as described by Freitas et al. (2020), with *impB* predicted to encode a type VI secretion protein. The pAR-0780 *optrA* region also contained the *erm*(A) resistance gene. The remaining pAR-0780 sequence was nearly identical (100% query coverage, 99.88% nucleotide sequence identity) to a 51,543 bp region of the 84,468 bp plasmid, pE508, a pheromone-responsive conjugative multiresistance plasmid recently characterized from *E. faecalis* strain E508 (ST256) (Figure 3). The pE508 plasmid was isolated from a swine fecal sample in China in 2015 and, in addition to *optrA* and *fexA*, is predicted to encode the aminoglycoside resistance gene *aac*(A)-*aph*(D) locus, and the tetracycline resistance genes *tet*(L) and *tet*(O/W/32/O) (Shang et al., 2019). Plasmid typing of pAR-0780 revealed a copy of *repA2* within the pAR-0780-pE508 common region that shared 99.8% nucleotide sequence identity with *repA2* from *E. faecalis* pTEF2 (accession AE016831) and belonged to the *rep9* plasmid family (Jensen et al., 2010; Zou et al., 2020). The pAR-0780-pE508 common region also contained the sex pheromone gene cluster, including *prgY*/*traB*, *prgZ*/*traC*, *prgR*, *prgA*/*sea1*, *prgB*/*asc10*, *prgU*, *prgC*, and genes associated with conjugal transfer, e.g., a conjugal transfer protein and type IV secretion system proteins (Shang et al., 2019; Zou et al., 2020). The complete pAR-0780 plasmid hybrid assembly (PacBio and Illumina) was deposited in GenBank under accession CP063981.

### Transferability of the *optrA*-Encoding Plasmid, pAR-0780

To assess the transferability of pAR-0780, filter mating experiments were performed using *E. faecalis* AR-0780 (LZD-R, MIC 8 μg/ml) as the donor and *E. faecalis* JH2-2 (fusidic acid-resistant and LZD-S, MIC 2 μg/ml) as the recipient. Selection of transconjugants that were LZD- and fusidic acid-resistant revealed pAR-0780 had a conjugation frequency of 1.1 × 10^–1^ (transconjugants per donor). Two transconjugants (LZD-R, 8 μg/ml and fusidic acid-resistant) were selected for confirmation of *optrA* transfer. Transconjugant 1 and transconjugant 2 were PCR-positive for the ∼1.4 kb *optrA* gene product (Supplementary Figure 1A). S1 nuclease-PFGE was used to linearize and size the plasmid content of the donor, recipient, and transconjugants (Supplementary Figure 1B). The recipient, JH2-2 contained no plasmids, but an ∼65 kb band corresponding to the pAR-0780 plasmid was present in both donor and recipient samples, indicating plasmid transfer.

## Discussion

Here we characterize DAP-R and LZD-R isolates from CDC’s Sentinel Surveillance collection of 708 enterococci collected during 2013–2016. While the number of DAP-R or LZD-R isolates was low, the presence of the emerging *E. faecium* ST736 associated with DAP-R, and the acquired LZD mechanism of resistance, *optrA*, with the propensity to efficiently spread via horizontal gene transfer raises concerns.

The rate of DAP-R *E. faecalis* was 0.5% and the rate of *E. faecium* was 4.9%. Resistance to DAP can occur through multiple pathways (Diaz et al., 2014; Wang et al., 2018; Prater et al., 2021), and all mechanisms have not been fully elucidated. While primary mutations in the LiaFSR system and subsequent mutations in the phospholipid metabolism enzyme, Cls, were identified for the majority of DAP-R isolates (15/19, 79%) in this collection, mutations previously associated with DAP-R were not identified in four isolates (one *E. faecalis*, ST40 and three *E. faecium*, ST78, ST1840, and ST1841). One novel mutation was identified in isolate 1603515, which contained an alanine to proline substitution in Cls (Cls^A20P^). While the contribution of this mutation to DAP-R is unknown, an alanine to aspartic acid substitution in Cls at that same position (Cls^A20D^) was previously characterized in DAP-R isolates (Wang et al., 2018).

Limited data are available on the clonal distribution of DAP-R enterococcal clinical isolates in the United States (Wang et al., 2014). Of the 19 DAP-R isolates (two *E. faecalis* and 17 *E. faecium*), WGS revealed the majority (71%, 12/17) of DAP-R *E. faecium* isolates belonged to the emerging clone, ST736. Daptomycin-resistant and VAN-resistant ST736 *E. faecium* were first identified in a hospital in New York (Wang et al., 2014), expanded throughout New York, and have been reported in Washington, Texas, and Maryland, as well as Canada, the Caribbean, Germany, and South America (Wang et al., 2018).

In the present collection of ST736 isolates, 7/12 (58%) were also VAN-R, with all isolates harboring *vanA*. These ST736 strains of *E. faecium* contain mutations in the LiaFSR system and sometimes subsequent mutations in the related phospholipid metabolism enzymes that predispose them to DAP resistance. *E. faecium* ST736 isolates are believed to share a unique genetic background that predisposes them to DAP-R and dissemination in the healthcare setting (Wang et al., 2018). Their growing geographic distribution in the United States and internationally has raised concerns about the global dissemination of this DAP-R clone (Wang et al., 2018). The DAP-R ST736 isolates characterized in this study were isolated from hospitalized patients from Iowa, Maryland, New York, Pennsylvania, and Washington. This is consistent with reports that ST736 is expanding in the United States.

All DAP-R ST736 isolates contained mutations in the LiaFSR system, LiaR^W73C^ and LiaS^T120A^, previously described in ST736 isolates, while a subset (5/12, 42%) contained subsequent mutations in Cls. Prior exposure to DAP while patients were hospitalized has been associated with acquisition of these subsequent mutations in Cls, along with higher DAP MICs than isolates with mutations in LiaFSR alone (Wang et al., 2018). A limitation of this work is the lack of exposure or treatment data to determine if this association exists for these surveillance isolates. The majority of ST736 DAP-R isolates with Cls mutations, 4/5 (80%), showed higher MICs (16 μg/ml) than those with LiaFSR mutations alone, which all displayed MICs of 8 μg/ml. All remaining DAP-R non-ST736 isolates had MICs of 8 μg/ml. One ST736 isolate, 1603370, that was selected for inclusion in this study because it was LZD-R, was found to contain the same LiaFSR mutations (LiaR^W73C^, LiaS^T120A^) seen in all ST736 isolates and was DAP-susceptible dose dependent (MIC of 4 μg/ml) according to CLSI interpretive guidelines (CLSI, 2021). Previous studies have also reported DAP-susceptible isolates with either LiaFSR or Cls mutations that alone are not sufficient to confer resistance to DAP, while the combination of mutations in both LiaFSR and Cls have been sufficient to confer DAP-R (Wang et al., 2018). A limitation of this study is that repeat or alternate antimicrobial susceptibility testing methods, e.g., gradient diffusion strips, were not performed and isolates near the MIC breakpoint for resistance, but without known DAP-R genotypes, could potentially be susceptible upon repeat testing or testing with an alternate method. In addition, only isolates determined to be resistant to DAP or LZD were sequenced; therefore, the number of DAP-susceptible *E. faecium* isolates from this collection that contain mutations in either LiaFSR or Cls that may predispose them to becoming resistant upon exposure to DAP is unknown.

Linezolid resistance was rare among the 2013–2016 Sentinel isolate collection, with only a single LZD-R *E. faecalis* isolate and two LZD-R *E. faecium* isolates identified. A recent survey of clinical *E. faecium* from the United States determined 30 LZD-R isolates harbored the G2576T 23S rRNA nucleotide mutation, instead of other acquired genes associated with LZD-R, such as *optrA*, *cfr*, or *poxtA* (Wardenburg et al., 2019). Both *E. faecium* isolates contained the common mutation associated with LZD resistance, the G2576T 23S rRNA mutation (Hasman et al., 2019). In addition, these *E. faecium* isolates belonged to the emerging healthcare-associated ST736 clone described above and contained the LiaR^W73C^ and LiaS^T120A^ mutations associated with DAP-R, including isolate 1603370 that was DAP-susceptible dose dependent, and 1603243, which is the only isolate in this study resistant to DAP, LZD, and VAN.

Long-read sequencing revealed the LZD-R *E. faecalis* isolate contained two copies of the ABC-F family ribosomal protection protein, *optrA*, one on the chromosome and one on an approximately 65 kb plasmid. Worldwide, there has been rapid emergence of LZD-R clinical and agricultural isolates carrying *optrA* since it was first report in 2015; however, the prevalence of *optrA* in the United States has remained low (Pfaller et al., 2017; Deshpande et al., 2018), which was consistent with this collection, where only a single *optrA-*harboring *E. faecalis* isolate was identified. To the best of our knowledge, all reports of *optrA* isolates collected in the United States have been plasmid-encoded (Pfaller et al., 2017; Deshpande et al., 2018; Tyson et al., 2018). Isolates containing two copies of *optrA* appear to be rare but have been described. For example, *E. faecalis* strain C25 isolated from a pig in China between 2016 and 2017, harbored chromosomal- and plasmid-encoded copies of *optrA*. Here we characterize the first U.S. clinical isolate, with both a chromosomal- and plasmid-encoded *optrA*. This VAN-S isolate was submitted to the CDC and FDA Antibiotic Resistance Isolate Bank, designated AR-0780, and is publicly available as part of the ‘‘Isolates with New or Novel Antibiotic Resistance’’ panel^7^.

The chromosomal copy of *optrA* is encoded on a recently characterized non-conjugative multiresistance Tn*554*-family transposon designated Tn*6674* and first characterized in the *E. faecalis* strain E1731, which was isolated in 2018 from a swine fecal sample in China. Subsequent analysis of publicly available sequence data by Freitas et al. (2020) revealed Tn*6674* has likely driven the spread of *optrA* among hospitalized patients (with examples from France, China, and Greece), healthy humans (with examples from China and Malaysia), and food-producing animals (with examples from China, Malaysia, and Tunisia) throughout 2012–2018. To the best of our knowledge this represents the first description of a clinical isolate carrying the chromosomal Tn*6674* transposon in the United States (Li et al., 2019; Freitas et al., 2020). While Tn*6674* was found to be non-conjugative, Li et al. (2019) detected circular intermediate forms of the transposon in *E. faecalis* strain E1731, indicating the transposon was active in E1731 and providing a potential mechanism for the dissemination of chromosomally-encoded *optrA* in enterococci. Tn*6674* not only encodes *optrA*, conferring resistance to oxazolidinones like LZD that are used to treat human enterococcal infections, but also encodes *fexA*, which confers resistance to phenicols that are used in food-producing animals (Li et al., 2019), presenting the concerning possibility of co-selection, maintenance, and spread of both resistance factors.

The plasmid encoding *optrA*, pAR-0780, also contained the antimicrobial resistance genes *erm(A)* and the *impB-fexA-optrA* segment, which was originally described in the first identified *optrA* plasmid, pE349, and various other plasmids identified in human and pig strains in China (He et al., 2016; Freitas et al., 2020). The pAR-0780 *optrA* region was > 99.9% identical to the *optrA* gene cluster previously described on plasmid p10-2-2 from an *E. faecalis* isolated from a pig in China (He et al., 2016). Additional plasmid-encoded *optrA* regions with similar gene arrangements and sequence identities have also been identified on plasmids from clinical isolates, including 739884 (China) and 912300 (United States), although the complete plasmid sequences for these isolates were not available (Deshpande et al., 2018). Similar to AR-0780’s chromosomal *optrA* region, the co-localization of *optrA* on pAR-0780 with other resistance genes, supports the transfer, co-selection, and persistence of *optrA* on both the chromosome and plasmid in this isolate when under selective pressure of phenicols and oxazolidinones. This again highlights the problem of antibiotic use selecting for resistance against multiple classes of antibiotics. Use of chloramphenicol may select for resistance not only to chloramphenicol but also to LZD.

The backbone of pAR-0780 was nearly identical to pE508, a conjugative multiresistance plasmid recently characterized from *E. faecalis* strain E508 (Shang et al., 2019). This shared region contained genes required for a highly efficient conjugation process referred to as the pheromone-responsive plasmid (PRP) transfer system. These PRPs are unique to enterococci and have transfer rates that reach or exceed 10^–1^ transconjugants per donor (or one transconjugant per 10 donor cells) *in vivo* and under laboratory conditions (Hirt et al., 2005; Sterling et al., 2020). pAR-0780 belonged to the *rep9*-type plasmid family, which have been characterized as sex-pheromone or PRPs (Jensen et al., 2010; Zou et al., 2020), and these plasmids have been recently associated with dissemination of *optrA* in clinical *E. faecalis* isolates in China (Shang et al., 2019; Zou et al., 2020). pAR-0780 had a high conjugation frequency of 1.1 × 10^–1^ (transconjugants per donor), which was 10.5% higher than the transfer frequency of 3.7 × 10^–2^ reported for pE508 (Shang et al., 2019).

pAR-0780 is a conjugative pheromone-responsive mosaic plasmid with high sequence similarity to plasmids belonging to animal isolates from diverse locations, suggesting pAR-0780 is the result of a horizontal gene transfer and/or a recombination event, potentially involving an animal reservoir. The use of long-read sequencing was critical in resolving the genetic landscape of AR-0780’s multiple copies of *optrA* and deciphering that the two copies were different *optrA* alleles. The chromosomal *optrA* on the Tn*6674* transposon and the copy of *optrA* encoded on the conjugative PRP were likely acquired during independent genetic events.

AR-0780 was isolated from a patient in the United States in 2015, the same year *optrA* was first reported (Wang et al., 2015). There are limited data available on the prevalence of LZD-R *optrA*- positive isolates in the United States, but work presented here, as well as other surveillance systems targeting clinical and animal isolates (Pfaller et al., 2017; Deshpande et al., 2018; Tyson et al., 2018) suggest *optrA* in the United States is rare. However, reports of rapid global dissemination of *optrA*-carrying enterococci, beyond the Asia-Pacific region (Bender et al., 2018; Deshpande et al., 2018; Sassi et al., 2019; Egan et al., 2020), as well as their location on active and highly efficient mobile genetic elements, presents the possibility that the prevalence of *optrA* in the United States is underestimated and national surveillance efforts to better understand *optrA* prevalence are needed.

Linezolid and daptomycin are important antibiotics for the treatment of infections caused by VRE. Resistance to these drugs was rare among the CDC’s 2013–2016 Sentinel Surveillance collection. This work found that a single DAP-R *E. faecium* clone, ST736, may be driving DAP-R resistance. We also identified a LZD-R isolate with *optrA* encoded not only on a chromosomal transposon, but also on a highly transmissible plasmid. This work highlights the importance of continued surveillance to monitor the emergence and spread of DAP-R and LZD-R clones and antimicrobial resistance mechanisms in the United States.

## Data Availability Statement

The datasets presented in this study can be found in online repositories. The names of the repository/repositories and accession number(s) can be found below: WGS short read data for all isolates in this study were submitted to the NCBI Sequence Read Archive (SRA) with the BioSample numbers SAMN16584109–SAMN16584128. The complete chromosome hybrid assembly (PacBio and Illumina) for AR-0780 was deposited in GenBank under BioProject accession number PRJNA523425, and short read data were submitted to the NCBI SRA under BioSample number SAMN11953790.

## Author Contributions

ASG, MK, and JDL conceived the project. ASG, JDL, LMS, WZ, and DC contributed to the design of experiments. LMS, WZ, DC, GM, TOE, VA, VAS, MS, and JP collected the data and performed the experiments. ASG, AGK, DC, and DB performed sequence analysis. ASG, AGK, LMS, and DC created the tables and figures. ASG led development of the manuscript. All authors contributed to drafting, revising, and approving the final submission.

## Conflict of Interest

LMS, TOE, and AGK were employed by the company Goldbelt C6, LLC. JP was employed by the company ASRT Incorporated. The remaining authors declare that the research was conducted in the absence of any commercial or financial relationships that could be construed as a potential conflict of interest.

## Publisher’s Note

All claims expressed in this article are solely those of the authors and do not necessarily represent those of their affiliated organizations, or those of the publisher, the editors and the reviewers. Any product that may be evaluated in this article, or claim that may be made by its manufacturer, is not guaranteed or endorsed by the publisher.

## Footnotes

1
https://www.applied-maths.com/

2
https://cge.cbs.dtu.dk/services/LRE-finder/

3
https://digitalinsights.qiagen.com

4
https://www.cdc.gov/hai/pdfs/labsettings/unified_pfge_protocol.pdf

5
https://pubmlst.org/organisms/enterococcus-faecalis

6
https://pubmlst.org/bigsdb?db=pubmlst_efaecium_isolates

7
https://wwwn.cdc.gov/ARIsolateBank/Panel/PanelDetail?ID=10
