# Supplementary figures and images for: Sentinel Surveillance Reveals Emerging Daptomycin-Resistant ST736 Enterococcus faecium and Multiple Mechanisms of Linezolid Resistance in Enterococci in the United States
Source: Front Microbiol. 2022 Feb 1;12:807398. doi: 10.3389/fmicb.2021.807398 (PMC8846945; doi:10.3389/fmicb.2021.807398)

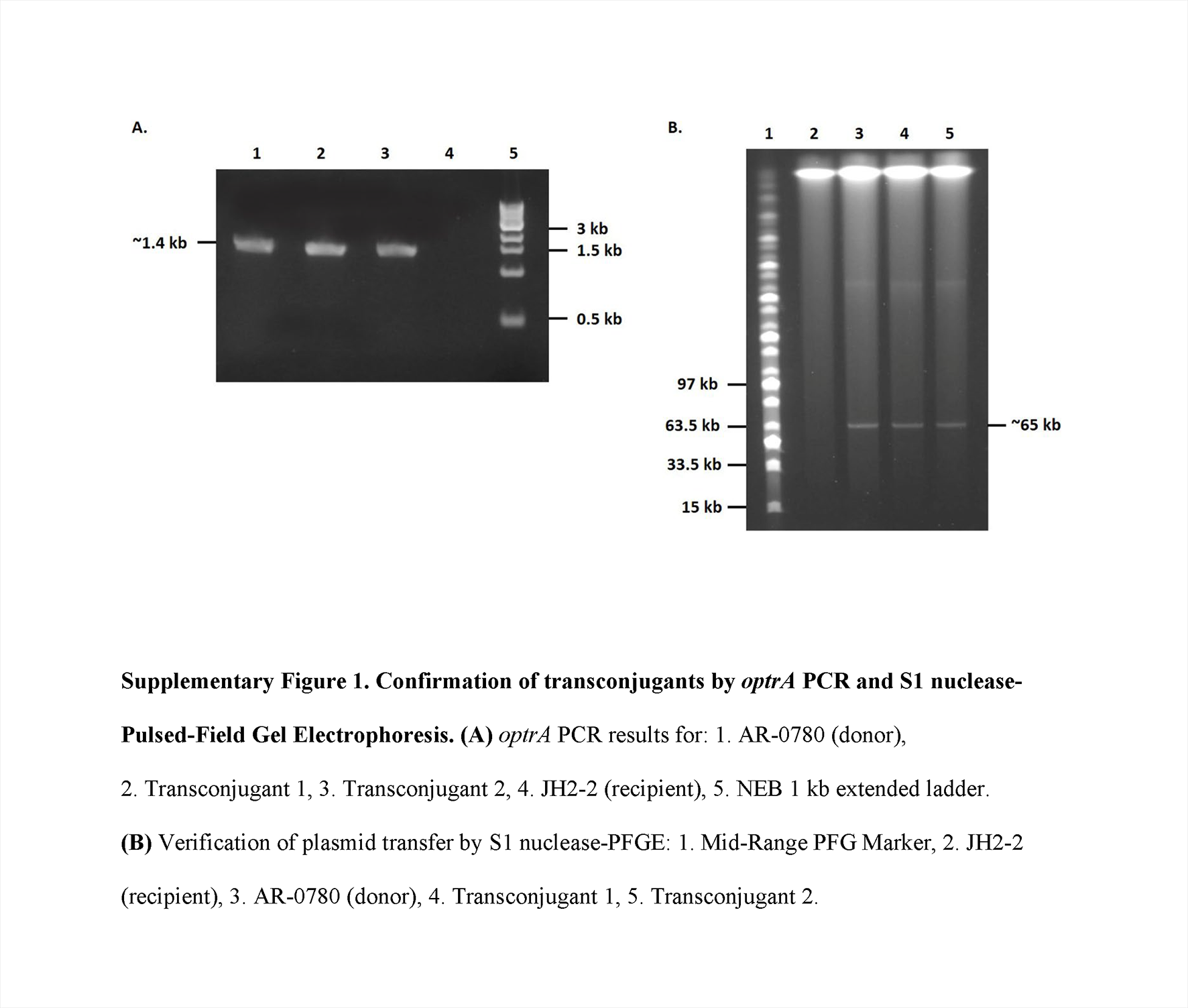

Supplement: Supplementary file 1 [file Image_1.tiff]
