# Supplementary material for: Sentinel Surveillance Reveals Emerging Daptomycin-Resistant ST736 Enterococcus faecium and Multiple Mechanisms of Linezolid Resistance in Enterococci in the United States
Source: Front Microbiol. 2022 Feb 1;12:807398. doi: 10.3389/fmicb.2021.807398 (PMC8846945; doi:10.3389/fmicb.2021.807398)
Supplement: Supplementary file 2 [file Table_1.docx]

**Supplementary Table 1.** Antimicrobial susceptibility profiles of *Enterococcus faecalis* and *Enterococcus faecium* CDC Sentinel Surveillance isolates with daptomycin and/or linezolid resistance.

|  | | | **BMD MIC µg/ml^a^** | | | | | | | | | | | | | | |
| --- | --- | --- | --- | --- | --- | --- | --- | --- | --- | --- | --- | --- | --- | --- | --- | --- | --- |
| **Isolate ID** | **BioSample Number** | **Organism ID** | **DAP** | **LZD** | **VAN** | **AMP** | **LVX** | **Q-D^b^** | **DOX** | **PEN** | **TEC** | **CHL** | **HLGR^c^** | **HLSR^c^** | **ERV** | **TGC** | **TZD** |
| 1603244 | SAMN16584113 | *E. faecalis* | 16 | 1 | 1 | 0.5 | 1 | 8 | 8 | 2 | 1 | 4 | + | - | 0.03 | 0.06 | 0.25 |
| 1603442 | SAMN16584119 | *E. faecalis* | 8 | 1 | 2 | 1 | 1 | 8 | 8 | 2 | 0.5 | 4 | - | - | 0.06 | 0.12 | 0.25 |
| AR-0780 | SAMN11953790 | *E. faecalis* | 1 | 8 | 1 | 1 | >8 | 4 | 0.9 | 2 | 0.5 | >32 | - | - | 0.03 | 0.06 | 1 |
| 1603019 | SAMN16584109 | *E. faecium* | 16 | 2 | >64 | >32 | >8 | 1 | 16 | >32 | >64 | 8 | - | - | 0.06 | 0.12 | 0.25 |
| 1603084 | SAMN16584110 | *E. faecium* | 8 | 2 | >64 | >32 | >8 | 1 | 16 | >32 | >64 | 16 | - | - | 0.06 | 0.12 | 0.5 |
| 1603162 | SAMN16584111 | *E. faecium* | >16 | 2 | >64 | >32 | >8 | 0.5 | 16 | >32 | >64 | 8 | - | - | >0.25 | 0.5 | 0.5 |
| 1603243 | SAMN16584112 | *E. faecium* | 8 | 16 | >64 | >32 | >8 | 0.5 | 1.9 | >32 | >64 | 8 | - | - | 0.06 | 0.12 | 2 |
| 1603274 | SAMN16584114 | *E. faecium* | 8 | 2 | >64 | >32 | >8 | 1 | 4 | >32 | >64 | 8 | - | + | 0.03 | 0.06 | 0.25 |
| 1603275 | SAMN16584115 | *E. faecium* | 8 | 1 | 1 | >32 | >8 | 0.5 | 4 | >32 | 1 | 4 | - | - | 0.25 | >0.50 | 0.25 |
| 1603296 | SAMN16584116 | *E. faecium* | 16 | 1 | >64 | >32 | >8 | 1 | 16 | >32 | >64 | 8 | - | - | 0.06 | 0.12 | 0.25 |
| 1603370 | SAMN16584117 | *E. faecium* | 4 | 8 | 0.5 | >32 | >8 | 0.5 | 16 | >32 | 1 | 8 | - | - | 0.06 | 0.12 | 1 |
| 1603389 | SAMN16584118 | *E. faecium* | 8 | 1 | 1 | 32 | >8 | 0.5 | 8 | 32 | 2 | 4 | - | - | 0.06 | 0.12 | 0.25 |
| 1603468 | SAMN16584120 | *E. faecium* | 8 | 1 | >64 | >32 | >8 | 0.5 | 16 | >32 | >64 | 4 | - | - | 0.06 | 0.06 | 0.25 |
| 1603514 | SAMN16584121 | *E. faecium* | 8 | 2 | 1 | >32 | >8 | 0.5 | 16 | >32 | 2 | 4 | - | + | 0.06 | 0.06 | 0.5 |
| 1603515 | SAMN16584122 | *E. faecium* | 16 | 2 | >64 | >32 | >8 | 0.25 | 16 | >32 | 16 | 4 | - | - | 0.03 | 0.06 | 0.5 |
| 1603630 | SAMN16584123 | *E. faecium* | 8 | 2 | 1 | >32 | >8 | 0.5 | 16 | >32 | 2 | 16 | - | - | 0.12 | 0.12 | 0.5 |
| 1603637 | SAMN16584124 | *E. faecium* | 8 | 2 | 1 | 8 | >8 | 4 | 1.9 | 32 | 2 | 8 | - | - | 0.06 | 0.06 | 0.5 |
| 1603688 | SAMN16584125 | *E. faecium* | 8 | 2 | 1 | >32 | >8 | 0.5 | 16 | >32 | 2 | 4 | - | - | 0.06 | 0.12 | 0.5 |
| 1704051 | SAMN16584126 | *E. faecium* | 16 | 2 | >64 | >32 | >8 | 1 | 16 | >32 | 64 | 8 | - | - | 0.12 | 0.25 | 0.5 |
| 1704054 | SAMN16584127 | *E. faecium* | 8 | 2 | 1 | >32 | >8 | 0.5 | 4 | >32 | 1 | 4 | - | - | 0.25 | >0.50 | 0.25 |
| 1704095 | SAMN16584128 | *E. faecium* | 8 | 1 | >64 | >32 | >8 | 1 | 4 | >32 | >64 | 8 | - | - | 0.06 | 0.06 | 0.25 |

The following antibiotics and ranges of concentration were included in the broth microdilution panels: daptomycin/DAP (0.25 – 16 µg/ml); linezolid/LZD (0.5 – 16 µg/ml); vancomycin/VAN (0.25 – 64 µg/ml); ampicillin/AMP (0.25 – 32 µg/ml); levofloxacin/LVX (0.25 – 8 µg/ml); quinupristin/dalfopristin/Q-D (0.12 – 8 µg/ml); doxycycline/DOX (2 – 32 µg/ml); penicillin/PEN (0.25 – 32 µg/ml); teicoplanin/TEC (0.12 – 64 µg/ml); chloramphenicol/CHL (2 – 32 µg/ml); gentamicin/GEN_500 (500 µg/ml), streptomycin/STR_1000 (1000 µg/ml); eravacycline/ERV (0.015 – 0.25 µg/ml); tigecycline/TGC (0.03 – 0.5 µg/ml); tedizolid/TZD (0.12 – 2 µg/ml).

*E. faecalis* are intrinsically resistant to quinupristin/dalfopristin

*E. faecalis* and *E. faecium* isolates are intrinsically resistant to aminoglycosides. High-level gentamicin resistance (HLGR) and high-level streptomycin resistance (HLSR) was tested for using 500 µg/ml and 1000 µg/ml of the antibiotic, respectively. A (+) implies resistance (i.e., no synergy between the aminoglycoside and a cell wall active agent and (–) implies susceptibility (i.e., synergy between the aminoglycoside and the cell wall active agent).
